# Prosomeric organization of the hypothalamus in an elasmobranch, the catshark *Scyliorhinus canicula*

**DOI:** 10.3389/fnana.2015.00037

**Published:** 2015-04-08

**Authors:** Gabriel N. Santos-Durán, Arnaud Menuet, Ronan Lagadec, Hélène Mayeur, Susana Ferreiro-Galve, Sylvie Mazan, Isabel Rodríguez-Moldes, Eva Candal

**Affiliations:** ^1^Centro de Investigaciones Biológicas, Department of Cell Biology and Ecology, University of Santiago de CompostelaSantiago de Compostela, Spain; ^2^Centre National de la Recherche Scientifique, Experimental and Molecular Immunology and Neurogenetics, University of OrleansUMR7355, Orleans, France; ^3^Centre National de la Recherche Scientifique, FR2424, Development and Evolution of Vertebrates Group, Sorbonne Universités – Université Pierre et Marie CurieRoscoff, France

**Keywords:** chondrichthyan, forebrain patterning, evolution, development, prosomeric model, *Shh*, *Nkx2.1*, Otp

## Abstract

The hypothalamus has been a central topic in neuroanatomy because of its important physiological functions, but its mature organization remains elusive. Deciphering its embryonic and adult organization is crucial in an evolutionary approach of the organization of the vertebrate forebrain. Here we studied the molecular organization of the hypothalamus and neighboring telencephalic domains in a cartilaginous fish, the catshark, *Scyliorhinus canicula*, focusing on *ScFoxg1a, ScShh, ScNkx2.1*, *ScDlx2/5*, *ScOtp,* and *ScTbr1* expression profiles and on the identification α-acetylated-tubulin-immunoreactive (ir), TH-ir, 5-HT-ir, and GFAP-ir structures by means of immunohistochemistry. Analysis of the results within the updated prosomeric model framework support the existence of alar and basal histogenetic compartments in the hypothalamus similar to those described in the mouse, suggesting the ancestrality of these subdivisions in jawed vertebrates. These data provide new insights into hypothalamic organization in cartilaginous fishes and highlight the generality of key features of the prosomeric model in jawed vertebrates.

## Introduction

Biological diversity emerges, at least in part, through changes in development. Organisms are different because their developmental process differ and, what is more, because their developmental process also evolve (Kutschera and Niklas, 2004; Müller, 2007; Medina et al., 2011). Thus, the understanding of the development of the vertebrate brain becomes fundamental to comprehend its structure and evolution. In this context, the hypothalamus has been both a central and elusive topic. The hypothalamus is a conserved integrative center that coordinates autonomic, endocrine, and limbic responses (Sarnat and Netsky, 1981; Kandel and Schwartz, 2001; Butler and Hodos, 2005). Its development, at the base of the vertebrate forebrain (prosencephalon), involves complex patterning processes dependent on different signaling events that converge at this point. It also undergoes a complex morphological deformation during development, which misleads its topological (*vs*. topographic) location (Shimamura et al., 1995; Puelles and Rubenstein, 2003; Puelles, 2009; Puelles et al., 2012). As a result, the hypothalamic organization remains a matter of debate (Figdor and Stern, 1993; Puelles and Rubenstein, 2003; Shimogori et al., 2010; Diez-Roux et al., 2011; Puelles et al., 2012). Cross-species comparisons can be important to resolve this issue, and an important effort to understand the underlying unity of hypothalamic embryonic and adult organization across vertebrates has been made recently (Shimogori et al., 2010; Domínguez, 2011; Morales-Delgado et al., 2011, 2014; Moreno et al., 2012; Domínguez et al., 2013, 2014; Herget et al., 2014).

The prosomeric model (Puelles and Rubenstein, 2003; Puelles et al., 2004, 2012; Medina, 2008; Puelles, 2009) has become a key reference in such comparative studies, since it offers a mechanistic paradigm of the vertebrate brain structure and organization. Initially based on analyses of amniotes, this model defines for the first time anatomical structures as developmental hierarchical units based on specification mechanisms that determine longitudinal and transverse axis orientation, segmental structure, transcription factor expression profiles and the emergence of differential histogenetic domains (Puelles and Rubenstein, 2003; Puelles, 2009; Martínez et al., 2012).

A major interest and novelty of this model is that it puts emphasis on developmental criteria (including topological relationships among certain morphological landmarks, regulatory gene expression patterns and signaling molecules). Testing their conservation across vertebrates is a powerful approach for the correct establishment of homologies between embryonic territories beyond amniotes (Puelles and Medina, 2002). The underlying notion is that formation of the vertebrate brain involves a conserved core of highly constrained, invariant mechanisms and genetic networks, which are the basis for homology establishment. This in no way excludes the emergence of diversifications through evolution, which are the source of the neuroanatomic diversity observed among vertebrates.

Latest updates of the model provide novel views on the organization of the rostral-most (secondary) prosencephalon, and its telencephalic and hypothalamic moieties (Puelles and Rubenstein, 2003; Pombal et al., 2009; Puelles et al., 2012). Detailed studies in different vertebrate groups are necessary to validate the model assumptions. Cartilaginous fishes or chondrichthyans are crucial in this task because they are among the most basal extant groups of gnathostomes (jawed vertebrates). Because of its phylogenetic position as the closest out-group to osteichthyans (the other major phylum of gnathostomes, which includes bony fish and tetrapods), chondrichthyans are essential to reconstruct gnathostome ancestral characteristics through comparisons with other vertebrate models. Here we studied the molecular histogenetic organization of the hypothalamus and directly adjoining territories of an elasmobranch representative of one of the most basal extant gnathostome lineage, the catshark *Scyliorhinus canicula*, and analyzed them under the updated prosomeric framework. We have integrated data from neuroepithelial specification codes (based on the expression of catshark orthologues of *Foxg1a*, *Shh*, *Nkx2.1*, *Dlx2/5*, *Otp,* and *Tbr1*), and from the distribution of α-acetylated-tubulin-immunoreactive (-ir) and TH-ir cell groups, neuron-fiber tracts (5-HT-ir) and glial-processes (GFAP-ir). In the search of conserved traits among jawed vertebrates, we compared our data in *S. canicula* with that obtained in murine models. Our analysis reveals a strikingly high degree in the conservation of hypothalamic histogenetic compartments between chondrichthyan and murine models. Furthermore, we identified some of the boundaries and confirmed some of the assumptions predicted by the prosomeric model. However, some differences and discrepancies also exist mainly concerning the neuroepithelial specification genetic codes of the basal hypothalamus (BHy). Similar studies are required in other basal species to figure out if these differences should prompt the model update or they are the consequence of shark specialization.

## Materials and Methods

### Phylogenetic Reconstructions

Sequence alignments of the sequences listed in Table S1 were constructed using the alignment editor Seaview 3.0 and the MUSCLE algorithm. *S. canicula* sequences were retrieved by tblastn searches in transcriptomic databases obtained by Sanger and Illumina sequencing of embryonic and adult cDNA libraries (stages 8–25 and mixed adult tissues). Maximum-likelihood trees were inferred using the PhyML program version 3, the LG-F+Γ12+I substitution model and the SPR algorithm. Posterior probabilities (PPs) supporting groupings were calculated using the aLRT algorithm implemented in PhyML and are displayed as percentages at the corresponding nodes. Only PP >80% are indicated. The trees were viewed and edited using Mega6.

### Experimental Animals

Some embryos of the catshark (lesser spotted dogfish; *S. canicula*) were supplied by the Marine Biological Model Supply Service of the CNRS UPMC Roscoff Biological Station (France) and the Estación de Bioloxía Mariña da Graña (Galicia, Spain). Additional embryos were kindly provided by the Aquaria of Gijón (Asturias, Spain), O Grove (Pontevedra, Spain) and the Aquarium Finisterrae (A Coruña, Spain). Embryos were staged by their external features according to Ballard et al. (1993). For more information about the relationship of the embryonic stages with body size, gestation and birth, see Table 1 in Ferreiro-Galve et al. (2010). Thirty-seven embryos from stages 12 to 31 were used in this study. Eggs from different broods were raised in seawater tanks in standard conditions of temperature (15–16^∘^C), pH (7.5–8.5) and salinity (35 g/L). Adequate measures were taken to minimize animal pain or discomfort. All procedures conformed to the guidelines established by the European Communities Council Directive of 22 September 2010 (2010/63/UE) and by the Spanish Royal Decree 53/2013 for animal experimentation and were approved by the Ethics Committee of the University of Santiago de Compostela.

### Tissue Processing

Embryos were deeply anesthetized with 0.5% tricaine methanesulfonate (MS-222; Sigma, St. Louis, MO, USA) in seawater and separated from the yolk before fixation in 4% paraformaldehyde (PFA) in elasmobranch’s phosphate buffer [EPB: 0.1 M phosphate buffer (PB) containing 1,75% urea, pH 7.4] for 48–72 h depending on the stage of development. Subsequently, they were rinsed in phosphate buffer saline (PBS), cryoprotected with 30% sucrose in PB, embedded in OCT compound (Tissue Tek, Torrance, CA, USA), and frozen with liquid nitrogen-cooled isopentane. Parallel series of sections (12–20 μm thick) were obtained in transverse and sagittal planes on a cryostat and mounted on Superfrost Plus (Menzel-Glasser, Madison, WI, USA) slides.

### Single and Double Immunohistochemistry on Sections and Whole Mounts

For heat-induced epitope retrieval, sections were pre-treated with 0.01 M citrate buffer (pH 6.0) for 30 min at 95^∘^C and allowed to cool for 20–30 min at room temperature (RT). Sections were then rinsed twice in 0.05 M Tris-buffered saline (TBS; pH 7.4) for 5 min each and incubated overnight with the primary antibody (rabbit anti-serotonin [anti-5-HT] polyclonal antiserum, DiaSorin, Immunostar, Hudson, WI, USA, diluted 1:5000; polyclonal rabbit anti-Sonic Hedgehog [anti-Shh], Sta. Cruz Biotechnology, Santa Cruz, CA, USA, diluted 1:300; polyclonal rabbit anti-glial fibrillary acidic protein [anti-GFAP], Dako, Glostrup, Denmark, diluted 1:500; and monoclonal mouse anti-tyrosine hydroxilase [anti-TH], Millipore, Billerica, MA, USA, diluted 1:500). Appropriate secondary antibodies [horseradish peroxidase (HRP)-conjugated goat anti-rabbit and anti-mouse, BIORAD, diluted 1:200] were incubated for 2 h at RT. For double immunohistochemistry (IHC) experiments, cocktails of primary antibodies were mixed at optimal dilutions and subsequently detected by using mixtures of appropriate secondary antibodies. Sections were rinsed in distilled water (twice for 30 min), allowed to dry for 2 h at 37^∘^C and mounted in MOWIOL 4-88 Reagent (Calbiochem, MerkKGaA, Darmstadt, Germany). All dilutions were made with TBS containing 15% donkey normal serum (DNS; Millipore, Billerica, MA, USA), 0.2% Triton X-100 (Sigma) and 2% bovine serum albumin (BSA, Sigma). Double IHC with primary antibodies raised in the same species was performed as described in Tornehave et al. (2000).

For whole mounts embryos were prepared as previously described in Kuratani and Horigome (2000) with minor modifications. After fixation with 4% PFA in 0.01 M PBS at 4^∘^C for 2 days, embryos were washed in 0.9% NaCl in distilled water, dehydrated in graded series of methanol solutions (50, 80, 100%) and stored at -20^∘^C. Samples to be stained were placed on ice in 2 mL of dimethyl sulfoxide (DMSO)/methanol (1/1) until they sank. Then, 0.5 mL of 10% Triton X-100/distilled water was added, and the embryos were incubated for 30 min at RT. After washing in 0.05 M TBS with 0.1% Triton X-100 (TST, pH 7.4) the samples were sequentially blocked using spin-clarified aqueous 1% periodic acid and 5% non-fat dried milk in TST (TSTM). Primary antibody (monoclonal mouse anti-α-acetylated-tubulin, Sigma, 1:1000) was diluted in TSTM containing 0.1% sodium azide for 2–4 days at RT with gently agitation on a shaking platform. The secondary antibody HRP-conjugated goat anti-rabbit, BIORAD, dilution 1:200 in TSTM) was incubated overnight. After a final washing in TST, the embryos were pre-incubated with 0.25 mg/mL diaminobenzidine tetrahydrochloride (DAB, Sigma) in TST with 2.5 mg/mL nickel ammonium sulfate for 1 h, and then allowed to react with DAB in TST containing 2.5 mg/mL nickel ammonium sulfate and 0.00075% H_2_O_2_ for 20–40 min at RT. The reaction was stopped using Tris-HCL buffered saline and specimens were post-fixed with 4% PFA overnight at 4^∘^C. Epidermis and mesodermic derivatives were carefully removed and specimens were rinsed in graded series of glycerol (25, 50, 75, and 100%) in order to directly observe the neural tube under the stereomicroscope.

### Controls and Specificity of the Antibodies

No immunostaining was detected when primary or secondary antibodies were omitted during incubations. Controls and specificity of anti-TH and anti-5-HT were performed as described in Pose-Méndez et al. (2014). The primary anti-α-acetylated-tubulin antibody has been shown to label early differentiated neurons and their processes in the embryonic nervous system (Piperno and Fuller, 1985; Chitnis and Kuwada, 1990). The polyclonal anti-Shh antibody (Santa Cruz Biotechnology Inc, CA, USA) was raised in rabbit against the amino acids 41–200 of Shh human protein. The *in situ* hybridization (ISH) results were similar to those obtained by IHC, and therefore validate the specificity of the anti-Shh antibody used here.

### *In Situ* Hybridization on Whole Mount Embryos and on Sections

We applied ISH for *ScFoxg1a*, *ScShh* (Compagnucci et al., 2013; Quintana-Urzainqui, 2013), *ScNkx2.1* (Quintana-Urzainqui et al., 2012; Quintana-Urzainqui, 2013), *ScDlx5* (Compagnucci et al., 2013; Debiais-Thibaud et al., 2013), *ScOtp* (Quintana-Urzainqui, 2013), *ScTbr1* (Quintana-Urzainqui, 2013), and *ScDlx2* (Quintana-Urzainqui et al., 2012; Compagnucci et al., 2013; Debiais-Thibaud et al., 2013; Quintana-Urzainqui, 2013) genes. These probes were selected from a collection of *S. canicula* embryonic cDNA library (mixed stages 9–22), constructed in pSPORT1, and submitted to high throughput EST sequencing. cDNA fragments were cloned in pSPORT vectors. Sense and antisense digoxigenin-UTP-labeled and fluorescein-UTP-labeled probes were synthesized directly by *in vitro* transcription using as templates linearized recombinant plasmid DNA or cDNA fragments prepared by PCR amplification of the recombinant plasmids. ISH in whole mount and on cryostat sections was carried out following standard protocols (Coolen et al., 2009). Briefly, sections were permeabilized with proteinase K, hybridized with sense or antisense probes overnight at 65^∘^C (in sections) or 70^∘^C (whole mount) and incubated with the alkaline phosphatase-coupled anti-digoxigenin and anti-fluorescein antibody (1:2000, Roche Applied Science, Manheim, Germany) overnight at 4^∘^C. The color reaction was performed in the presence of BM-Purple (Roche). Control sense probes did not produce any detectable signal.

### Inhibition of the *Shh* Pathway

Inhibition of the Shh pathway was performed by *in ovo* injection of the pharmacological inhibitor cyclopamine in order to test whether, as in osteichthyans, the initiation of *ScNkx2.1* expression in the forebrain is dependent on Shh. First, 200 μL of a solution containing 1x PBS, 500 μM cyclopamine and 5% DMSO were injected through the shell of stage 15–16 *S. canicula* eggs. This solution was replaced by the same volume of 5% DMSO in 1x PBS for control embryos. The eggs were maintained for 3 days in oxygenated sea water at 17^∘^C, with viabilities higher than 90%. Embryos reached stage 18 in these conditions. They were dissected, fixed in PFA 4%, dehydrated and stored in methanol 100% prior to ISH.

### Image Acquisition and Analysis

Light field images were obtained with an Olympus BX51 microscope equipped with an Olympus DP71 color digital camera. *In toto* embryos were analyzed in the Olympus SZX12 stereomicroscope fitted to an Olympus DP12 color digital camera. Photographs were adjusted for brightness and contrast and plates were prepared using Adobe Photoshop CS4 (Adobe, San Jose, CA, USA).

## Results

### Identification of Catshark Orthologues of the Genes Studied

**FIGURE 1:**
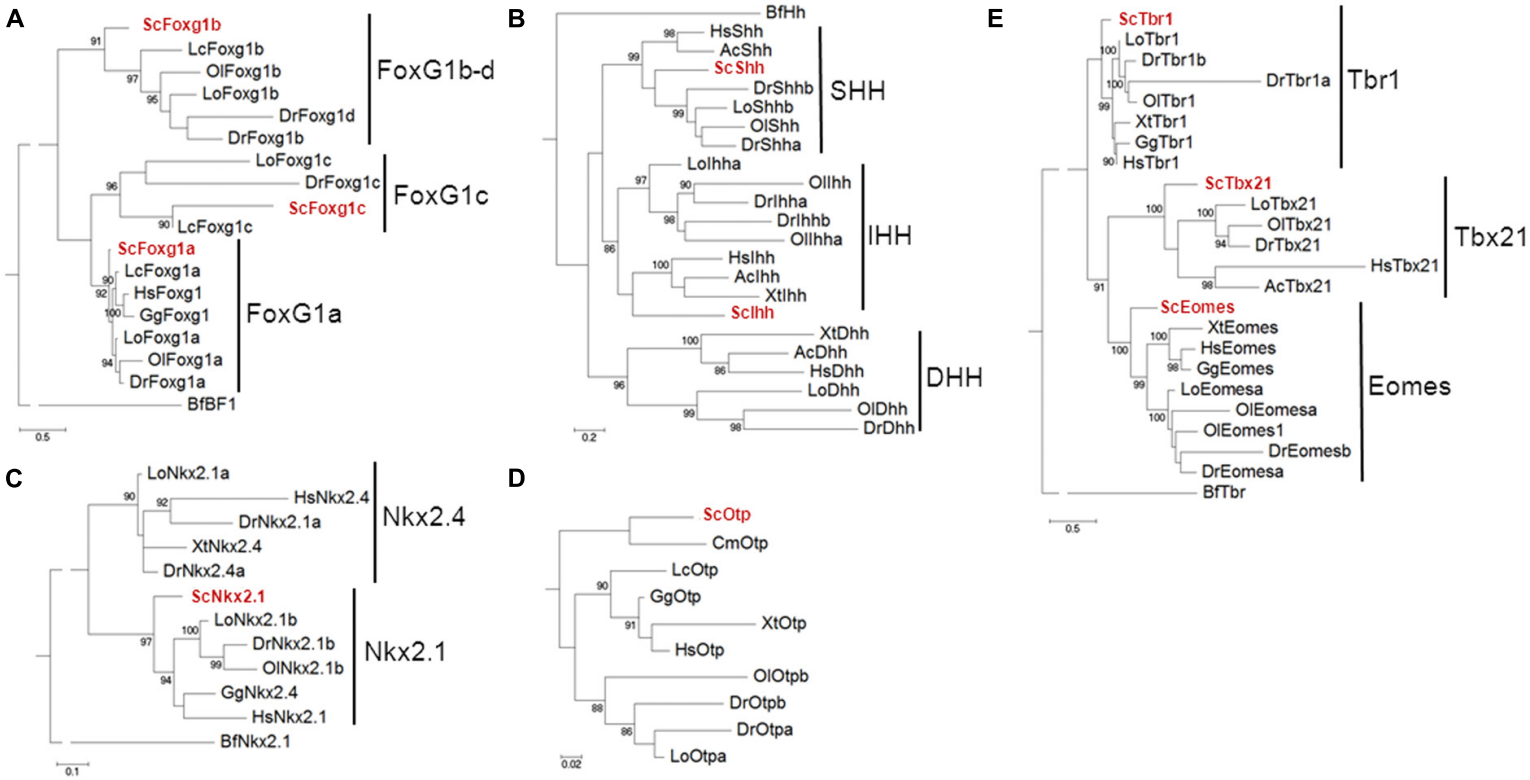
**Phylogenetic analysis of the *Scyliorhinus canicula* genes analyzed in this study**. Phylogenetic trees for the Foxg1, Hedgehog, Nkx2.1/Nkx2.4, Otp and Tbr1/Tbx21/Eomes families are shown in **(A–E)** respectively. The number of substitutions per site is indicated at the bottom of each tree, on the left. *S. canicula* genes are displayed in red. Abbreviations used: Hs, *Homo sapiens* (human); Gg, *Gallus gallus* (chick); Ac, *Anolis carolinensis* (anole lizard); Xt, *Xenopus tropicalis* (African clawed frog); Lc, *Latimeria chalumnae* (coelacanth); Lo, *Lepisosteus oculatus* (spotted gar); Ol, *Oryzias latipes* (medaka); Dr, *Danio rerio* (zebrafish); Cm, *Callorhinchus milii* (elephant shark); Sc, *Scyliorhinus canicula* (catshark or lesser spotted dogfish); Bf, *Branchiostoma floridae* (amphioxus).

Exhaustive phylogenetic characterizations of the catshark *Dlx* gene repertoire have been previously published (Debiais-Thibaud et al., 2013), confirming the identity of *ScDlx2* and *ScDlx5*. In order to unambiguously identify the catshark orthologues of *Foxg1*, *Shh*, *Nkx2.1*, *Otp,* and *Tbr1*, we conducted systematic phylogenetic analyses of the corresponding vertebrate gene families, including all the vertebrate classes derived from duplications of a single ancestral chordate orthologue (**Figure 1**). In each case, phylogenies were constructed from alignments containing deduced amino acid sequences of all paralogous sequences retrieved from catshark transcriptomic databases and from a representative sampling of actinopterygians and sarcopterygians. The trees were rooted using a *Branchiostoma floridae* sequence, except in the case of Otp which could not be found in the amphioxus Ensembl database. In the case of Foxg1, three strongly supported classes (posterior probability or PP > 90%), each containing a catshark and several osteichthyan sequences, were retrieved, highlighting for the first time the presence of three gnathostome Foxg1 classes (**Figure 1A**). These classes were termed Foxg1a, Foxg1b, and Foxg1c, respectively. One coelacanth and several actinopterygian sequences, but no amphibian or amniote sequence, were found in the Foxg1b and Foxg1c classes, suggesting a loss of their representatives in tetrapods. We focused the expression analysis on *ScFoxg1a*, the catshark orthologue of the only *Foxg1* gene retained in all major gnathostome lineages including tetrapods. The tree topology obtained for the Hedgehog family confirmed the presence of the three gnathostome classes, corresponding to the Indian Hedgehog, Desert Hedgehog and Sonic Hedgehog classes already reported in osteichthyans, and confirmed *ScShh* as the representative of the latter (**Figure 1B**). Concerning the Nkx2.1/Nkx2.4 family, a single catshark gene could be identified and it was unambiguously assigned to the Nkx2.1 class based on the strongly supported grouping of its deduced amino acid sequence with teleost, chick, and human Nkx2.1 sequences (PP = 97%; **Figure 1C**). This gene is therefore referred to as *ScNkx2.1* hereafter. A single catshark Otp related sequence, termed ScOtp, could be found and as expected, it clustered with the elephant shark sequence annotated as Otp in the reconstruction shown in **Figure 1D**. Finally, the Tbr1, Tbx21, and Eomes classes were retrieved with high statistical support (PP = 83, 100, and 99%, respectively) within the Tbr1/Tbx21/Eomes family. Each class contained a single catshark sequence at the expected position, allowing an unambiguous identification of the *ScTbr1* gene analyzed in this study (**Figure 1E**).

### Preliminar Considerations Concerning Vertebrate Segmental Prosencephalic Organization

**FIGURE 2:**
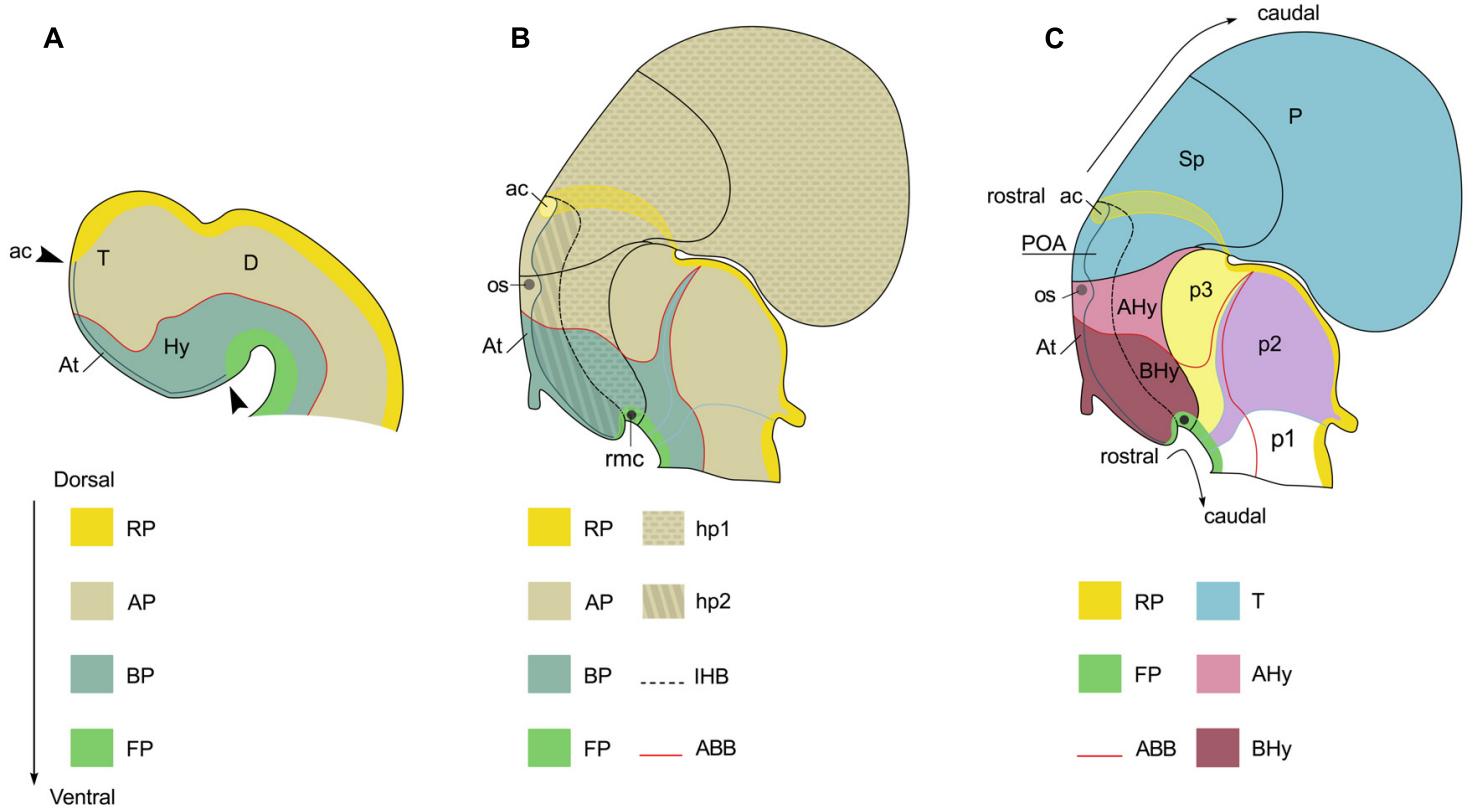
**Squematic representations of the prosencephalon of early **(A)** and late **(B,C)** mouse embryo to show correspondence of longitudinal and tranverse domains in the secondary prosencephalon under the updated prosomeric model**. Domains in **(A)** are illustrated according to Figure 1.1C in Martínez et al. (2012). Domains in **(B,C)** are illustrated according to Figure 8.5B in Puelles et al. (2012). **(A)** Longitudinal domains in early embryos. The arrowheads mark both the dorso-caudal and ventro-caudal limits of the acroterminal territory (At). This territory is considered the rostral-most domain of the neural tube. The dorso-caudal limit of the At can be identified caudal to the anterior commissure. **(B)** Longitudinal and transverse organization in late embryos. **(C)** Segmental organization of the secondary prosencephalon according to the prosomeric model. For abbreviations, see list.

The organization of the shark hypothalamus has been analyzed in the framework of the updated prosomeric model (Puelles et al., 2012). **Figure 2** summarizes the general architecture of the hypothalamus in mouse according to the updated prosomeric model (Puelles et al., 2012). This model is mainly inspired in murine data though it is usually assumed that it can be extrapolated to all vertebrates because it also integrates information from other vertebrates (Puelles and Rubenstein, 2003; Pombal et al., 2009; Puelles, 2009). Indeed, this model represents a useful developmental and comparative framework since it makes use of concepts, nomenclature and topological references that can be used across different vertebrate species.

The prosomeric model establishes that hypothalamus and telencephalon are part of the secondary prosencephalon, which is understood as a segmental unit at the rostral-most point of the neural tube, the hypothalamus being located ventral to the telencephalon and rostral to the diencephalon (see **Figure 2A**).

The model also postulates that the rostral-most point of the brain, referred as the acroterminal region (At), lies at the rostral border of the secondary prosencephalon. This region is restricted to the frontal border of the neural tube where left and right alar and basal plates meet. This border expands dorso-ventrally from the rostral-most roof plate (which is telencephalic) to the rostral-most floor plate (which is hypothalamic). Thus, every structure classically considered being dorsal or ventral to these points (see arrowheads in **Figure 2A**), should be considered as caudal in this framework. Of note, the anterior commissure, located in the rostral-most roof plate, is a clear landmark of both the dorso-ventral and rostro-caudal axis (Puelles et al., 2012; see also **Figures 2A,B**).

The secondary prosencephalon presents two true segments rostro-caudally arranged (**Figure 2B**): hp2 (rostral or terminal) and hp1 (caudal or peduncular). Each segment harbors telencephalic and hypothalamic derivatives (**Figures 2B,C**). However, the telencephalon harbors only roof and alar plates while the hypothalamus harbors alar, basal, and floor plate derivatives. The existence of these segments is supported by several genes differentially expressed in the rostro-caudal axis, the location of commissures in the roof and floor plates (anterior and retromammillary commissures, respectively), and the course of important tracts [medial forebrain bundle (mfb); lateral forebrain bundle (lfb); and fornix (fx)] running by a common path at the rostral border of hp1, through alar and basal plates. These data, in turn, support the existence of an intersegmental boundary that separates terminal and peduncular subdivisions of both telencephalon and hypothalamus, which is referred as the intrahypothalamic boundary (IHB; **Figures 2B,C**). Caudally, the secondary prosencephalon limits with the diencephalon at the hypothalamic diencephalic border (HDB), another intersegmental limit among hp1 and p3, though it should be noticed that part of the caudal limit of the secondary prosencephalon does correspond to the telencephalon (Puelles et al., 2012; see also **Figure 2C**).

The model considers the adult hypothalamic organization arranged in different histogenetic territories defined by neuroepithelial specification codes and radial units (Puelles and Medina, 2002; Puelles et al., 2012). These codes reveal that telencephalon and hypothalamus belong to different histogenetic territories being the preoptic area (POA) the unique terminal territory of the telencephalon (**Figure 2C**). Of note, the POA also harbors the anterior commissure (Puelles et al., 2012; see also **Figures 2B,C**).

### *ScFoxg1a* Expression

**FIGURE 3:**
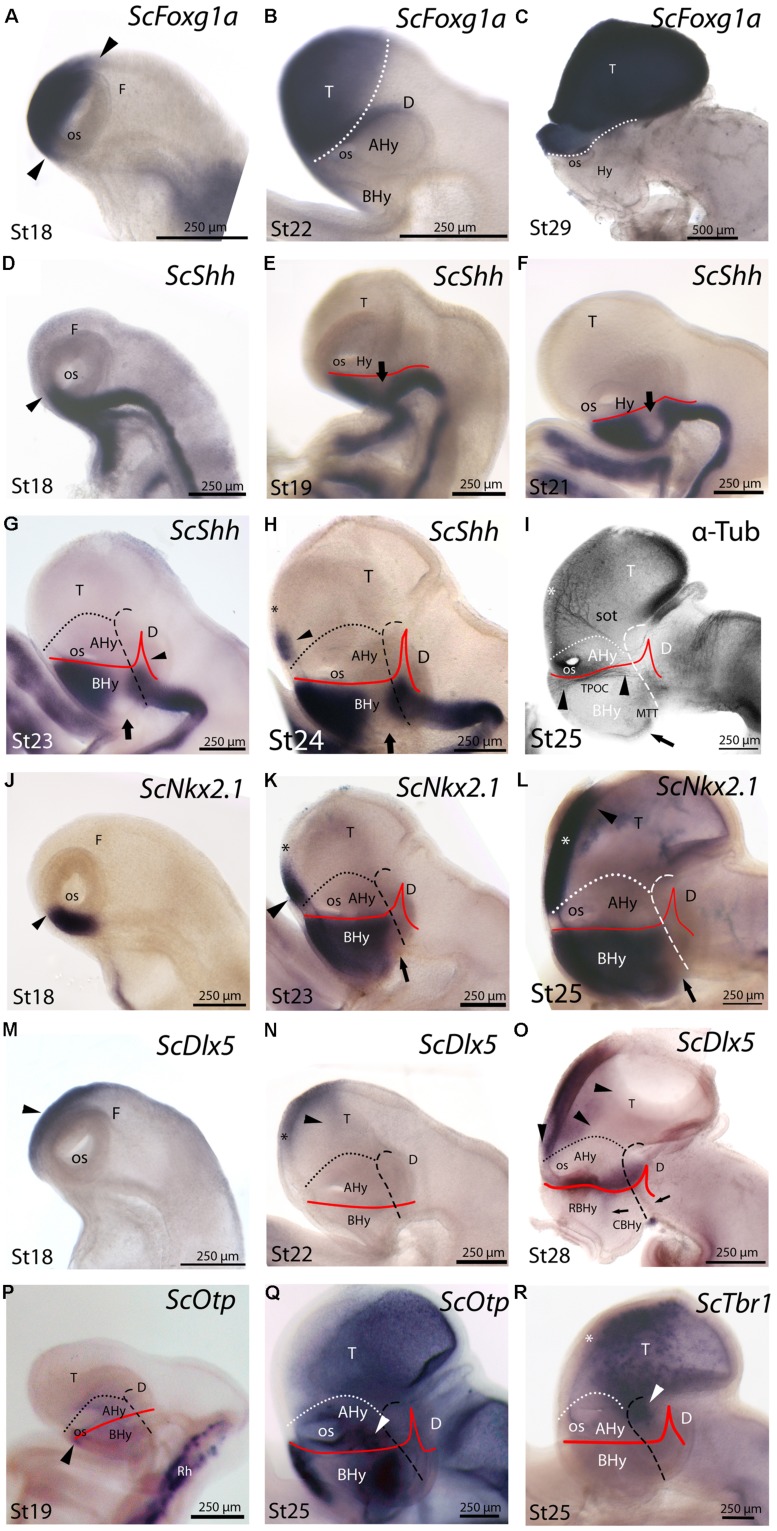
**Regionalization of the hypothalamus and neighbor territories in embryos of *S. canicula* from stages 18–29 based on the expression pattern of *ScFoxg1a***(A–C)**, *ScShh***(D–H)**, *ScNkx2.1***(J–L)**, *ScDlx5***(M–O)**, *ScOtp***(P,Q)**, *ScTbr1***(R)** genes and α-acetylated-tubulin immunoreactivity **(I)**.** In all panels, dotted lines define the hypothalamo-telencephalic boundary (HTB), dashed lines indicate the caudal border of the secondary prosencephalon and red lines indicate the ABB. **(A–C)** *ScFoxg1a* expression in the secondary prosencephalon at indicated stages. The arrowheads in **(A)** mark the caudo-dorsal and rostro-ventral limit of *ScFoxg1a* expression. **(D–H)** *ScShh* expression at the indicated stages. The arrowhead in **(D)** marks the rostral-most point of *ScShh* expression in the forebrain. The arrows in **(E–H)** indicate the downregulation of *ScShh* expression in the hypothalamus. The arrowhead in **(G)** points to the developing zli. The arrowhead in **(H)** points to a novel domain in the telencephalon. The asterisk in **(H)** marks the prospective territory of the anterior commissure. **(I)** Anti-α-acetylated-tubulin IHC to show three sets of tracts at stage 25. These tracts are classically referred as sot, TPOC and MTT. The asterisk indicates the territory of the developing anterior commissure. The arrowheads point to the longitudinal TPOC. The arrow points to the rostral-most extension of the MTT. **(J–L)** *ScNkx2.1* expression at the indicated stages. The arrowhead in **(J)** points to the rostral-most point of *ScNkx2.1* expression at stage 18, which was restricted to a short longitudinal domain ventrally to the optic stalk. The arrow in **(K,L)** points to a small *ScNkx2.1*-negative domain at the most caudo-ventral BHy. The asterisk in **(K,L)** marks the prospective territory of the anterior commissure. The arrowhead in **(K,L)** points to a domain in the telencephalon that spread rostro-caudally. **(M–O)** *ScDlx5* expression at the indicated stages. The arrowheads in **(M,N)** indicate *ScDlx5* expression in the olfactory placode and the anterior part of the telencephalon. The asterisk in **(N)** indicates the prospective territory of the anterior commissure. The arrowheads in **(O)** point to the ventral and caudal expansion of *ScDlx5* expression in the telencephalon. This domain was fairly continuous with a longitudinal band of *ScDlx5* over the ABB. The arrows in **(O)** point to *ScDlx5*-expressing domains that spread into the BHy. **(P,Q)** *ScOtp* expression at the indicated stages. The arrowhead in **(P)** indicates a restricted domain of *ScOtp* expression ventrally located with respect to the optic stalk. The expression of *ScOtp* in the hypothalamus was faint compared to that of the Rh. The white arrowhead in **(Q)** points to *ScOtp* expression in the AHy. Two additional *ScOtp*-expressing domains were observed in the BHy. **(R)** *ScTbr1* expression at stage 25 was found in part of the telencephalon and at the dorsal-most part of the rostral diencephalon (white arrowhead). The asterisk indicates the prospective territory of the anterior commissure. For abbreviations, see list.

In mice, *Foxg1* is one of the earliest transcription factors expressed specifically in the part of the neural plate that gives rise to the telencephalon and it remains expressed throughout the telencephalon during embryonic development (see Manuel et al., 2011). In an attempt to discriminate telencephalic and underlying hypothalamic domains throughout *S. canicula* development, we have analyzed the expression of *ScFoxg1a* in the developing nervous system of this species. At stage 18, *ScFoxg1a* expression was found in the dorsal-most portion of the secondary prosencephalon including the optic cup, extending from the level of the optic stalk (which is located rostrally, within the At) up to a caudal point in the roof plate, which has been tentatively identified as the dorsal border between the telencephalon and the diencephalon (**Figure 3A**). At stage 22, *ScFoxg1a* was observed in the telencephalon and in the nasal part of the optic cup (**Figure 3B**). The expression in the telencephalon was maintained until late stages of development (**Figure 3C**), which allowed identifying the border between the telencephalon and the hypothalamus.

### *ScShh* Expression

**FIGURE 4:**
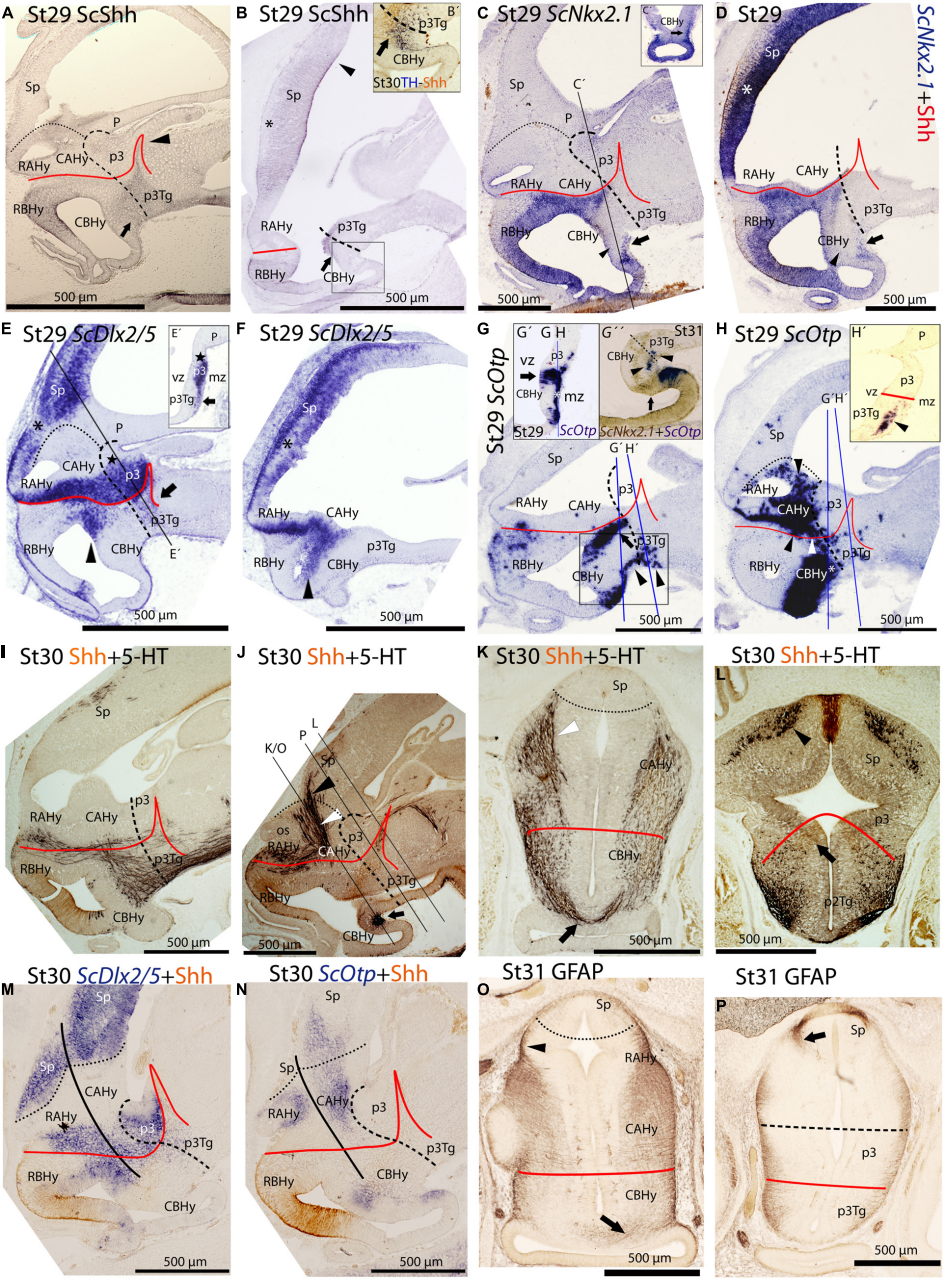
**Regionalization of the shark hypothalamus from stages 29–31 based on immunoreactivity to Shh **(A,B,B′,D,I–N)**, TH **(B′)**, 5-HT **(I–L)**, GFAP **(O, P)** and expression of *ScNkx2.*1 **(C,C′,D,G″)**, *ScDlx2/5***(E,E′,F,M)** and *ScOtp***(G,G′, G″, H, H′, N)** genes by means of single immunohistochemistry (IHC; **A,B,O,P)**, double IHC **(B′,I–L)**, single ISH **(C,E,E′,F,G, G′,H,H′)** and/or combined with IHC **(D,M,N)** on sagittal **(A,B,B′,C–G,G″,H–J,M,N)** or transverse sections **(C′,E′,G′,H′,K,L,O,P)**.** Image in **(G″)** results from the overlapping of two parallel sections respectively hybridized with *ScOtp* and *ScNkx2.1* probes. Color for *ScNkx2.1* was digitally converted to brown to ease comparison. Dotted lines define the hypothalamo-telencephalic boundary (HTB), dashed lines indicate the caudal border of the secondary prosencephalon, red lines indicate the ABB and continuous black lines represent the path followed by 5-HT-ir fibers. **(A)** Shh-immunoreactivity was observed in the most caudo-ventral part of the CBHy (arrow) and in the RBHy. The arrowhead points to the zli. **(B)** Shh-immunoreactivity was observed in the telencephalon (arrowhead) beyond the territory of the anterior commissure (asterisk). The arrow points to Shh-immunoreactivity in the caudal-most CBHy. **(B′)** Detail of the squared area in **(B)** to show Shh- and TH-immunoreactivity in its most caudo-ventral part (arrow). **(C,D)** *ScNkx2.1* expression was observed in the hypothalamus and telencephalon. A detail of a transverse section at the level indicated in **(C)** is shown in **(C′)**. The arrows in **(C,C′,D)** point to *ScNkx2.1*-expressing cells in the mantle zone of the most caudo-ventral CBHy. The arrowheads in **(C,D)** indicate a wedge-shaped domain lacking *ScNkx2.1* expression. The asterisk in **(D)** indicates the territory of the anterior commissure. **(E,F)** *ScDlx2/5* expression was observed in p3 and in the secondary prosencephalon. A detail of a transverse section at the level indicated in **(D)** is shown in **(D′)**. The arrow in **(E,E′)** points to *ScDlx2/5*-expressing cells in the mantle of the p3Tg. The star in **(E,E′)** indicates the prospective territory of the PThE. The black asterisks in **(E,F)** indicate a gap of *ScDlx2/5* expression in the telencephalon. The arrowheads in **(E,F)** point to *ScDlx2/5* expression in the BHy. **(G,H)** *ScOtp* expression in the hypothalamus. Details in **(G′,H′)** correspond to transverse sections at the levels indicated in **(G,H)**. Detail in **(G″)** correspond to the squared area in **(G)**. The arrows in **(G,G″)** point to the ventricular domain expressing *ScOtp* in the caudal CBHy. The black arrowheads in **(G,G″, H′)** point to *ScOtp*-expressing cells in the mantle of the most caudo-ventral part of CBHy and p3Tg. The arrow in **(G″)** indicates a domain expressing *ScNkx2.1* alone. The white asterisks in **(G′,H)** indicate *ScOtp*-expressing cells in the mantle zone. The black arrowhead in **(H)** points to *ScOtp*-expressing cells in the AHy and the white arrowhead in **(H)** points to *ScOtp*-expressing cells between the alar and basal domains.** (I–L)** Double Shh- and 5-HT-immunoreactivity. **(K,L)** correspond to trasverse sections at the level indicated in **(J)**. 5-HT-ir fibers in **(I)** are observed in the basal plate of the secondary prosencephalon. In the rostral hypothalamus such fibers coursed among RAHy and RBHy, and were located dorsally to Shh-immunoreactivity **(I,J)**. Note the presence of 5-HT-ir fibers in the Sp **(I,J,L)**. The white arrowhead in **(J,K)** points to 5-HT-ir fibers that course in the rostral CAHy. The black arrowhead in **(J,L)** points to 5-HT-ir fibers in the telencephalon. The arrow in **(J,K)** points to 5-HT-ir fibers decussating in the CBHy. The arrow in **(L)** points to the faint Shh immunoreactivity in the zli. **(M,N)** Shh IHC combined with *ScDlx2/5* expression **(M)** or *ScOtp* expression **(N)**. A gap of expression is observed between the rostral and caudal domains of *ScDlx2/5* and *ScOtp* expression, which appeared to coincide with the path followed by 5-HT-ir fibers. **(O)** GFAP-ir processes at the level shown in **(J)**. The arrowhead points to the GFAP-ir processes among RAHy and Sp. The arrow points to the GFAP-ir processes in the CBHy. **(P)** GFAP-ir processes at the level shown in **(J)**. The arrow points to GFAP-ir processes in the Sp. For abbreviations, see list.

*ScShh* expression was detected during gastrulation (stage 12) in the caudal midline of the embryo (data not shown). At stage 14, during early neurulation, it has been detected in the axial mesoderm of the notochord and the prechordal plate and in the ectoderm of the caudal midline (data not shown). After the closure of the neural tube (stage 17), the signal was detected as a ventral longitudinal continuous band that extends from the caudal end of the spinal cord to the At of the forebrain, roughly at the level of the optic stalk (**Figure 3D**). As in other vertebrates (Shimamura et al., 1995), the expression of *ScShh* can be used to define the alar-basal boundary (ABB; **Figures 3E–H**). At stage 19, *ScShh* expression became downregulated in the forebrain to progressively give rise to a caudal and a rostral domain (arrow in **Figures 3E–H**). The narrow transverse and dorsally directed stripe of *ScShh*-expressing cells within the caudal domain was identified as the developing zona limitans intrathalamica (zli; arrowhead in **Figure 3G**). The rostral border of the *ScShh* caudal domain, in turn, was somewhat extended rostral to the HDB (Puelles et al., 2012), which at this stage was identified as the point where the neural tube expands to acquire the distinctive shape of the ventral hypothalamus. Therefore, the BHy appeared to be divided in three domains: two positive for *ScShh* (one rostral and other caudal) and one (intermediate) negative for *ScShh* (arrow in **Figures 3G,H**; see also Figure 5H in Compagnucci et al., 2013). Of note, the dorsal border of the rostral domain (presumably corresponding to the ABB) seems to codistribute with α-acetylated-tubulin-immunoreactive (-ir) longitudinal tracts (arrowheads in **Figure 3I**). At stage 24 (**Figure 3H**), a new domain emerged within the telencephalon. This short domain (arrowhead in **Figure 3H**) extended from a region located dorsally to the optic stalk without reaching the prospective territory of the anterior commissure (that can be identified at early development by means of α-tubulin-immunoreactivity; asterisk in **Figures 3H,I**). A clear gap of expression was observed between this telencephalic domain and the rostral hypothalamic one (**Figure 3H**). The telencephalic domain was located medially while the hypothalamic one also expanded laterally (not shown). From stage 27 onward the zli expanded dorsally toward the roof plate (arrowhead in **Figure 4A**). At stage 29 the medio-lateral histologic organization of the developing walls of the forebrain become more evident. As in previous developmental stages, Shh immunoreactivity was clearly identified in the basal plate of the diencephalon entering the caudo-ventral part of the BHy (arrow in **Figures 4A,B,B′**) and in the rostro-dorsal part of the BHy (**Figures 4A,B**), so that a clear negative gap of Shh-immunoreactivity occupied most of the caudal BHy (CBHy; **Figures 4A,B**) and part of the rostral BHy (RBHy). In the telencephalon, Shh-immunoreactivity expanded caudally beyond the prospective territory of the anterior commissure (arrowhead in **Figure 4B**; compare with **Figure 3H**). Of note, from late stage 30 onward, Shh-immunoreactivity is downregulated in the CBHy and basal diencephalon, except in the zli (data not shown).

### *ScNkx2.1* Expression

The expression of *ScNkx2.1* was first detected at stage 18 in the rostro-ventral portion of the forebrain, in a longitudinal band which extended ventral to the optic stalk (arrowhead in **Figure 3J**). At stage 23 *ScNkx2.1* was expressed in most of the BHy (**Figure 3K**). Differently from *ScShh*, *ScNkx2.1* delimited the ABB even in the CBHy (**Figure 3K**; compare with **Figure 3G**), though a small gap of expression was observed within the caudo-ventral part of the BHy (arrow in **Figure 3K**). A second domain emerged in the telencephalon at this stage (arrowhead in **Figure 3K**). This domain was restricted to the rostral-most portion of the telencephalon and extended from a region located dorsally to the optic stalk to the prospective territory of the anterior commissure (asterisk in **Figure 3K**). A clear gap of expression was observed between the telencephalic and the hypothalamic domains. At stage 25 (**Figure 3L**), as in previous developmental stages, *ScNkx2.1* expression was lacking in a small domain located within the caudo-ventral BHy (arrow in **Figure 3L**). This region seems to fit with the rostral border of a basal α-acetylated-tubulin-ir tract [see mammillo-tegmental tract (MTT) in **Figure 3I**]. In the telencephalon, *ScNkx2.1* expression became caudally expanded beyond the prospective territory of the anterior commissure (asterisk in **Figure 3L**). At stage 29, as in previous developmental stages, *ScNkx2.1* was observed throughout most of the BHy, except in a small wedge-shaped domain within the caudo-ventral BHy (arrowhead in **Figures 4C,D**). Groups of *ScNkx2.1*-expressing cells in the caudo-ventral portion of the hypothalamus were observed along the marginal zone (arrows in **Figures 4C,C′,D**). In the telencephalon, *ScNkx2.1* expression expanded beyond the territory it occupied at previous developmental stages (asterisk in **Figure 4D**). Of note, Shh immunoreactivity was overlapping with *ScNkx2.1* expression beyond the anterior commissure (**Figure 4D**).

**FIGURE 5:**
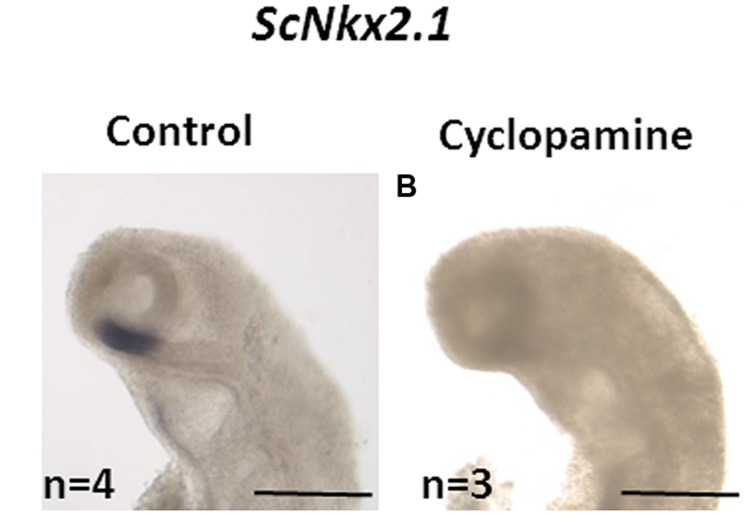
**Loss of *ScNkx2.1* early expression following cyclopamine treatment**. Lateral views of the cephalic region of stage 18 catshark embryos following whole-mount *in situ* hybridization (ISH) with the *ScNkx2.1* probe. Control **(A)** and cyclopamine-treated **(B)** embryos as described in Section “Materials and Methods” are shown. The number (n) of embryos used is indicated in each case. The signal is lost in the latter. Scale bars: 500 μm.

In order to test whether, as in osteichthyans, the initiation of *ScNkx2.1* expression in the forebrain is dependent on Shh, we used *in ovo* injections of the Shh inhibitor cyclopamine. All control embryos (*n* = 4) exhibited the expected *ScNkx2.*1 signal in the rostral-most and ventral-most portion of the forebrain (**Figure 5**). This signal was lost in all embryos dissected following cyclopamine treatment (*n* = 3), supporting the conclusion that Shh signaling is required for the initiation of *ScNkx2.1* expression in *S. canicula* as in osteichthyans.

### *ScDlx2/ScDlx5* Expression

We analyzed the expression of *ScDlx5* from stage 18 onward and the expression of *ScDlx2* from stage 29 onward. Fairly identical results were observed with both markers in the brain of *S. canicula* from stage 29 onward, so we use *ScDlx2/5* at these stages to refer indistinctly to both.

General features of *ScDlx5* expression and detailed profiles in the developing branchial arches have been previously described from stage 15 to stage 27 in Compagnucci et al. (2013) and from stage 15 to stage 25 in Debiais-Thibaud et al. (2013). We revisited these data focusing on the developing forebrain. At stage 18, *ScDlx5* expression was found in the most anterior part of the neural tube (**Figure 3M**; compare with **Figure 3A**; see also Figure 5C1 in Debiais-Thibaud et al., 2013). From stage 21 to 25, *ScDlx5* becomes mostly restricted to the anterior-most part of the telencephalon and to the olfactory placodes (**Figure 3N**; see also Figure 4G in Compagnucci et al., 2013 and Figure 5C’1 in Debiais-Thibaud et al., 2013). However, at later stages (**Figure 3O**), *ScDlx5* expression spread caudally and ventrally (arrowheads in **Figure 3O**) and reached the rostral-most portion of the optic stalk (see also Figure 9C in Debiais-Thibaud et al., 2013). This domain was fairly continuous with a longitudinal band of *ScDlx5* that crossed through the hypothalamus over the ABB (compare with **Figures 3H,L**) and entered p3 (**Figure 3O**). Therefore, this domain delineates the ABB along the hypothalamus. Of note, the longitudinal domain appeared to codistribute with α-acetylated-tubulin tracts (arrowheads in **Figure 3I**). Although both *ScDlx5* domains were continuous, a wedge-shaped area of reduced signal intensity was observed between the dorsal (telencephalic) and the ventral (hypothalamic-diencephalic) domains (**Figure 3O**). Two bands of cells were additionally observed, which were ventrally located with respect to the longitudinal domain (arrows in **Figure 3O**). One was located at its caudal end and spread ventral-ward at the rostral end of p3. The other spread perpendicularly to the ABB from the caudo-dorsal part of the BHy up to the rostral hypothalamus (**Figure 3O**). Of note, this *ScDlx5*-expressing domain appeared to delineate *ScShh* expression in the rostral hypothalamus (compare with **Figure 3H**). This pattern became more patent at stage 29 (**Figures 4E,F**). At this stage, *ScDlx2/5*-expression was observed in the telencephalon (subpallium). The telencephalic domain is almost continuous at medial levels (black asterisk in **Figure 4F**) while a clear gap of expression was observed at the level of the anterior commissure in parasagittal sections (black asterisk in **Figure 4E**). *ScDlx2/5* expression was also observed in a longitudinal domain outlining the ABB, which crossed through the alar hypothalamus (AHy) and entered p3 (**Figure 4E**). A wedge-shaped negative domain separated the telencephalic and hypothalamic-diencephalic domains (**Figure 4E**). A transverse band of non-ventricular *ScDlx2/5*-expressing cells was observed extending ventral-ward from the alar plate (arrow in **Figures 4E,E′**), along the rostral-most p3Tg. This domain appeared to overlap with the *ScShh*-expressing domain from which the zli emerged (compare with **Figure 4A**). As observed previously, an additional domain (arrowhead in **Figures 4E,F**) cut across the BHy perpendicularly to the ABB. This pattern was maintained until late stages of development (**Figure 4M**).

### *ScOtp* Expression

At stage 19, *ScOtp* signal was detected in the rhombencephalon and in the rostral-most and ventral-most portion of the optic stalk (arrowhead in **Figure 3P**) though a faint *ScOtp* expression was also observed in part of the BHy and in the AHy. At stage 25, three domains of *ScOtp* expression were observed in the forebrain. The rostral one was restricted to the rostral-most region of the forebrain, expanding ventrally from the optic stalk along the RBHy without reaching the prospective neurohypophysis. The second domain abutted the HDB at the intersection with the ABB and spread from this region up to the rostro-ventral hypothalamus. The third domain overlaid the ABB from the optic stalk up to the alar p3 and was poorly stained compared to the other two domains (arrowhead in **Figure 3Q**). From stage 28 onward these three domains were respectively identified in the RBHy (in **Figure 4G**), in an arched domain that spread from the CBHy (arrow in **Figures 4G,G′**) and in the AHy (black arrowheads in **Figure 4H**). In the BHy, a small domain containing *ScNkx2.1* alone was identified caudal to the *ScOtp*-expressing domain (arrow in **Figure 4G**). A novel domain of non-ventricular scattered *ScOtp-* expressing cells was also detected entering p3Tg from the most caudo-ventral part of the BHy (arrowheads in **Figures 4G,G″,H′**). Scattered *ScOtp*-expressing cells were observed between the alar and basal domains of the caudal hypothalamus (white arrowhead in **Figure 4H**). In the AHy, *ScOtp*-expressing cells were only observed at parasagittal levels (**Figure 4H**; compare with **Figure 4G**). Of note, non-ventricular *ScOtp*-expressing cells appeared to codistribute with the longitudinal band of *ScDlx2/5* expression over the ABB (**Figure 4H**; compare with **Figure 4F**). *ScOtp*-expressing cells were also observed in the telencephalon (data not shown). This pattern is maintained until late stages of development. At stage 30 (**Figure 4N**), a gap of *ScOtp* expression was observed at parasagittal levels that divide the AHy in rostral and caudal domains.

### *ScTbr1* Expression

*ScTbr1* signal was detected at stage 25 in dispersed cells that spread the telencephalic vesicle (**Figure 3R**), except for a rostral domain that extended from the optic stalk to the prospective territory of the anterior commissure (asterisk in **Figure 3R**). Of note, α-acetylated-tubulin-ir tracts reaching the telencephalon seem to define the boundary between telencephalic *ScTbr1* positive and negative domains (see sot in **Figure 3I**; compare with **Figure 3R**). *ScTbr1* expression was additionally observed in the dorsal most part of the alar p3, abutting the *ScDlx5* domain that entered p3 (**Figure 3R**; compare with **Figure 3O**). At stage 29, the extension of the *ScTbr1*-expressing domain became decreased in the telencephalic vesicle (not shown).

### 5-HT + Shh Immunoreactivity

Anti-5-HT immunoreactivity was coanalyzed with anti-Shh immunoreactivity at stage 30 to better understand the segmental organization of different fiber bundles, which in turn contributes to the understanding of the organization of the hypothalamus. Immunoreactivity for both markers was examined at stage 30 when the first 5-HT-ir fibers reach the telencephalon (Carrera et al., 2008). Positive fibers were observed coursing parallel to the ABB (**Figure 4I**). Besides, some fibers were detected ascending throughout the AHy (white arrowhead in **Figures 4J,K**) toward the telencephalon (black arrowhead in **Figures 4J,L**) from the caudal part of the hypothalamus. This pathway seemed to concur with negative domains for *ScDlx2/5* and *ScOtp* genes at the same developmental stage (**Figures 4M,N**). A group of decussating fibers was detected close to the most caudo-ventral region of the hypothalamus (arrow in **Figures 4J,K**) that coincides with the caudal-most and ventral-most domain of *ScNkx2.1* in the BHy (**Figure 4J**; compare with **Figures 4C,D**) and also with the rostral end of the ventral-most α-acetylated-tubulin-ir tract at stage 25, that could represent a pioneering tract, the mammillo-tegmental tract (MMT in **Figure 3I**). Of note, from stage 30 onward, Shh immunoreactivity was only observed in the rostral portion of the zli but not in the caudo-ventral part of the BHy nor the diencephalic basal plate (**Figure 4J** and arrow in **Figure 4L**).

### GFAP Immunoreactivity

Glial fibrillary acidic protein immunoreactivity was also analyzed in the forebrain at stage 31. Radial and longitudinal GFAP-ir processes were detected through the whole forebrain (**Figures 4O,P**). Ascending fibers to the telencephalon were detected in a similar pathway to that described above for 5-HT (black arrowhead in **Figure 4O**; compare with white arrowhead in **Figure 4K**). Some GFAP-ir processes were also observed in the same point where 5-HT-ir fibers decussate in the caudo-ventral part of the BHy (arrow in **Figure 4O**; compare with **Figure 4K**). GFAP-ir processes were also observed in the subpallium (arrow in **Figure 4P**).

## Discussion

### Alar Hypothalamus

**FIGURE 6:**
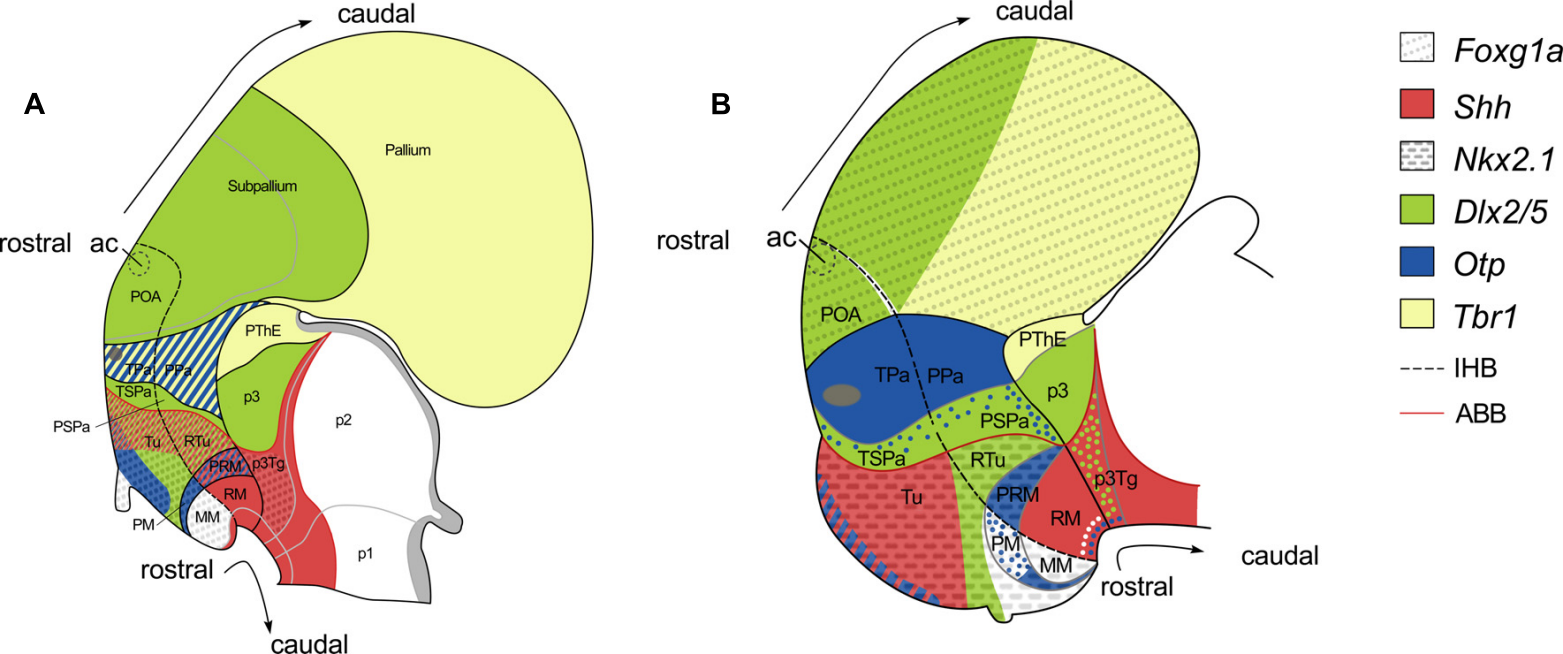
**Schematic representation of various gene expression patterns in the forebrain of mouse **(A)** and the rostral diencephalon and hypothalamus of *S. canicula***(B)**, and their correspondence with the updated prosomeric model (see text for details)**. Patterns in **(A)** are illustrated according to Figures 8.9 and 8.16 in Puelles et al. (2012). Patterns in **(B)** were mapped according to present results. Expression of *Nkx2.1* and *Otp* genes in the telencephalon has not been represented. White, green and blue dots represent non-ventricular *ScNkx2.1*-, *ScDlx2/5*-, and *ScOtp*-expressing cells, respectively. For abbreviations, see list.

According with the updated prosomeric model (Puelles et al., 2012; see also **Figure 6A**) the AHy, together with the telencephalon, are the rostral-most regions of the alar plate. The AHy is located ventral to the *Foxg1*-expressing telencephalon, dorsal to the BHy (which is characterized by the expression of *Nkx2.1* in its whole extension except in its caudal-most portion), and rostral to the alar p3 (characterized by the complementary expression of *Dlx* and *Tbr1* genes). Within the territory delimitated by the above-mentioned genes, two longitudinal (dorso-ventrally arranged) histogenetic domains are defined based on the complementary expression of *Dlx* and *Otp* genes. The dorsal-most domain is termed paraventricular domain (Pa), and expresses *Otp* but not *Dlx* genes. The ventral-most is the subparaventricular domain (SPa), which expresses *Dlx* genes but not *Otp*. *Dlx* is expressed beyond the HDB in the alar p3. The alar HDB is defined by the clear-cut expression among genes restricted to the AHy (*Sim1*, *Otp)* or to the alar p3 (*Lhx9*, *Arx*, *Olig2* among others). The alar p3, besides, includes the prethalamic eminence (PThE) and express genes in a complementary manner (PThE: *Tbr1*, *Lhx9*, *Gdf10*; alar p3: *Dlx* genes, *Arx*, *Gsh2*). Moreover, according with the prosomeric model both, Pa and SPa domains, present terminal (hp2) and peduncular (hp1) domains (TPa, PPa; TSPa, PSPa respectively). However these subdomains cannot be genetically identified without additional markers. Thus, the AHy presents at least four different histogenetic domains although some work on the development of hypothalamic peptidergic cells point to much more subdivision (**Figure 6A**; see also Morales-Delgado et al., 2011, 2014; Puelles et al., 2012).

In the shark, we have studied the expression of *ScFoxg1a*, *ScShh*, *ScNkx2.1*, *ScDlx2/5*, *ScOtp,* and *ScTbr1*, which allow us to identify an AHy harboring Pa-like and SPa-like histogenetic domains and their boundaries. The early expression territories of *ScFoxg1a, ScNkx2.1* and *ScOtp/ScTbr1* define the dorsal, ventral and caudal limits of the AHy, respectively (**Figures 3B,K,Q,R**; see also **Figure 6B**). Thus, *ScFoxg1a* was expressed from early stages of development in the presumptive telencephalon (**Figure 3C**), leading to the identification of the telencephalon-AHy border (see also **Figure 6B**). *ScNkx2.1* was expressed in most of the BHy (**Figure 3L**) and delineates the ABB (see also **Figure 6B**). The caudal border of the AHy could be delimited at stage 25 by the caudal-most expression of *ScOtp* at parasagittal levels (**Figure 3Q**; see also **Figure 6B**). Both *ScOtp* and *ScDlx5* were expressed from stage 25 onward within the AHy, though the intensity of the expression increases in late stages of development. At stage 29, both *ScOtp* and *ScDlx2/5* were observed in the AHy. Of note, two mutually exclusive histogenetic domains dorso-ventrally arranged could be readily observed at ventricular levels (**Figures 4E,H**; see also **Figure 6B**), though non-ventricular *ScOtp*-expressing cells were also observed within the *ScDlx2/5* domain. These observations allowed us to identify the TPa/PPa-like and the TSPa/PSPa-like domains (**Figure 6B**). The former, *ScOtp*-expressing one, abutted a *ScTbr1* domain that occupied the dorsal-most part of p3 (the PThE; **Figure 6B**), which is also negative for *ScDlx2/5* (compare with **Figures 4E,E′**). The latter, *ScDlx2/5*-expressing one, as in mouse, was continuous with the transverse *ScDlx2/5*-expressing domain in p3 (**Figures 4E** and **6B**).

In the mouse, and using markers homologous to those studied here, termino-peduncular compartments can be differentiated only by the late expression of *Tbr1* in the mantle of PPa (Puelles et al., 2012). While *ScTbr1* marker did not reveal rostro-caudal differences in the shark, we discerned these compartments by means of the identification of the mfb, which coursed caudally to the IHB (see below).

### Basal Hypothalamus

The BHy is the rostral-most territory of the basal and floor plates. It is located ventrally to the AHy and rostral to the basal p3 (p3Tg; **Figure 6A**). The BHy is characterized by the expression of *Nkx2.1* in its whole extension with the exception of its caudal-most region. It harbors three longitudinal domains dorso-ventrally arranged: tuberal/retrotuberal region (Tu/RTu); perimammillary/periretromammillary region (PM/PRM) and mammillary/retromammillary region (MM/RM). These domains harbor terminal and peduncular parts separated by the IHB. The peduncular domains are distinctly referred in this basal region with the prefix “retro” while the terminal lack prefix. In the mouse (**Figure 6A**), the Tu/RTu compartment is characterized by the expression of *Dlx5* although it also presents subdomains expressing *Shh* (in part of the Tu and the whole RTu); the PM/PRM is characterized by the expression of *Nkx2.1* and *Otp* although *Shh* is also expressed in the PRM; the MM only expresses *Nkx2.1* (being the caudal and ventral-most portion of the *Nkx2.1* domain) while the RM express *Shh* and other genes in a complementary manner to *Nkx2.1* (see Morales-Delgado et al., 2011, 2014; Puelles et al., 2012). The basal HDB is defined by the clear-cut expression among genes restricted to the caudal part of the hypothalamus (*Pitx2*, *Lhx5* among others) or to the p3Tg (*Nr5a1*, *Nkx6.1*; Puelles et al., 2004, 2012; Medina, 2008).

In the shark, the analysis of the expression of *ScShh*, *ScNkx2.1*, *ScDlx2/5,* and *ScOtp* highlighted the presence of Tu/RTu-like, PM/PRM-like and MM/RM-like histogenetic domains within the BHy. However, the relative organization of some of these expression territories differed between the catshark and mammals. While *ScNkx2.1* expression territories were well established in the BHy from early stages of development, the other markers analyzed here showed a more dynamic pattern through development. We chose to focus on stage 29 for the interpretation of these data, all studied markers exhibiting a strong expression at this stage, with sharp boundaries.

In mouse, according with the prosomeric model, the *Nkx2.1* territory in the basal plate located dorsal to the *Otp*, is considered to represent the Tu/RTu domain. *Dlx* genes are widely expressed in this domain (**Figure 6A** and Puelles and Rubenstein, 2003), though some tuberal areas lack *Dlx* gene signal (Morales-Delgado et al., 2011). Accordingly, in *S. canicula*, the BHy located dorsal to the *ScOtp*-expressing domain was interpreted as the Tu/RTu-like domain (**Figure 6B**). Within this territory, *ScOtp* was expressed in a restricted stripe at the most rostro-dorsal part of the basal plate spreading ventral-ward from the ABB without reaching the neurohypophysis (see **Figures 3Q and 6B**). *ScShh* overlapped with this domain, extended caudally and abutted a *ScDlx2/5*-expressing domain that extended from the ABB up to the neurohypophysis. Therefore, in sharks, the Tu/RTu-like domain would be composed by four subdomains: a rostro-dorsal subdomain coexpressing *ScShh*, *ScOtp* and *ScNkx2.*1; a second subdomain expressing *ScShh* and *ScNkx2.1*; a third subdomain expressing *ScDlx2/5* and *ScNkx2.1;* and a ventral subdomain expressing *ScNkx2.1* alone (compare **Figures 4C,D**; see also **Figure 6B**). As in mouse, and with the markers studied here, it was not possible to establish a clear distinction between the Tu-like and the RTu-like compartment. It is noteworthy that the Tu/RTu-like compartment has a different histogenetic identity to that described in the mouse. On one hand, *ScShh* was down-regulated in a large portion of the BHy. On the other, *ScDlx2/5* expression appeared much more restricted than in mouse, and different subdomains could be delineated, including a domain expressing *ScNkx2.1* alone in midsagittal sections (**Figures 4C,D**; see also **Figure 6B**). In mouse, the single domain in the BHy that contains *Nkx2.1* expression alone was identified as the MM region. The *ScNkx2.1* expressing region ventral to *ScDlx2/5* is unlikely to correspond to the MM for two reasons. First, it is not the only compartment where we detect the expression of *ScNkx2.1* alone. Indeed, in mid-sagittal sections, a small domain can be observed ventral to *ScOtp*, which expressed *ScNkx2.1* alone (**Figures 4G″** and **6B**). Second, the presence of a MM-like domain at this location would imply the interruption of longitudinal compartments (MM-like located dorsally to PM-like) and the redefinition of terminal domains within the BHy. Since it has been previously reported that, in mouse, some tuberal areas lack *Dlx* gene signal (Morales-Delgado et al., 2011), the most parsimonious interpretation implies that this *ScDlx2/5*-lacking region belongs to the Tu/RTu-like area and that *ScDlx2/5* cannot be used to identify the ventral border of the Tu/RTu-like domain, at least in mid-sagittal sections.

According to the prosomeric model, in mouse, adjacent to the Tu/RTu area, a distinct PM/PRM histogenetic domain exists in which *Otp* expression is selectively found. In *S. canicula*, *ScOtp* expression was observed in an arched domain that spread from the CBHy at the ABB/HDB junction and entered the rostral hypothalamus (**Figures 4G″** and **6B**). At its caudal-most portion, this domain seems to express *ScOtp* only in the ventricular zone (asterisk in **Figures 4G′,H**; see also **Figure 6B**), while in its rostral-most portion *ScOtp* is mainly expressed on mantle cells. This *ScOtp*-expressing domain (including either ventricular or mantle cells) was therefore interpreted as a PM/PRM-like domain, where the PRM-like domain is likely to correspond to caudal *ScOtp* expression in cells at the ventricular zone, and the PM-like would mainly correspond to rostral *ScOtp* expression in mantle cells.

As in mouse, a small domain containing *ScNkx2.1* alone was identified ventral to the *ScOtp*-expressing domain (**Figures 6A,B**). This domain abutted a *ScShh*-expressing domain which expanded caudally through the diencephalic basal plate (**Figure 6B**). Based on these expression similarities, the *ScNkx2.1* and *ScShh*-expressing territories were respectively identified as the MM-like and the RM-like domains. The caudal border of the RM-like domain abutted a transverse band of non-ventricular *ScDlx2/5*-expressing cells at the p3Tg (**Figures 4A,E**; see also **Figure 6B**).These *ScDlx2/5*-expressing cells are just ventral to the *ScDlx2/5*-expressing domain in the alar p3, supporting their assignment to p3Tg. Furthermore, these cells are in the same position that Pax6-ir cells in the caudal posterior tuberculum of the shark, described by Ferreiro Galve (2010) at equivalent developmental stages. Similar diencephalic basal plate *Dlx*-expressing cells have not been described in the mouse but *Dlx2-* and *Pax6*-expressing cells have been described in the basal plate of zebrafish as belonging to the preglomerular complex (p3Tg), suggesting that their presence may be an ancestral characteristic of jawed vertebrates lost in mammals (Ishikawa et al., 2007; Vernier and Wullimann, 2008). Accordingly to this, the HDB in the basal plate lie rostral to non-ventricular *ScDlx2/5*-expressing cells in p3Tg.

### Posterior Tuberculum

The shark hypothalamus and the posterior tuberculum have been analyzed before, mainly under neurochemical and topographical approaches. The posterior tuberculum in chondrichthyans extends caudally from the posterior recess (or mammillary recess). This recess lies just at the caudal and ventral border of *ScNkx2.1* expression in the MM-like domain and the rostral and ventral border of *ScShh* expression in the RM-like domain (**Figures 4A,C,D**; see also **Figure 6B**). A posterior tuberculum harboring TH-ir cells has been classically related to the hypothalamus (Smeets, 1998) although modern studies addressed it as belonging to p3Tg (Carrera, 2008; Carrera et al., 2008, 2012; Ferreiro-Galve et al., 2008; Quintana-Urzainqui et al., 2012). Our present genoarchitectonic analysis suggests that the bigger part and rostral-most located portion of TH-ir cells and fibers belongs to the RM-like (*ScShh*-expressing) domain while the caudal-most located portion of these TH-ir cells and fibers belongs to p3Tg (**Figure 4B′**). Thus, the rostral-most portion of TH-ir cells should be understood as hypothalamic. This configuration seems to fit parsimoniously with the prosomeric model under the light of the following facts. TH-ir ascending fibers from the rostral posterior tuberculum to the telencephalon seem to arise from these TH-ir hypothalamic groups (compare with Figures 4A,B in Carrera et al., 2012). As discussed below, these ascending TH-ir tracts seem to respect and follow the intersegmental boundary between hp2 and hp1 as proposed by the updated prosomeric model (Puelles et al., 2012), since they course in the most rostral part of hp1. Furthermore, equivalent cells in the posterior tuberculum and their ascending fibers to the telencephalon seem to exist across different vertebrate species (Vernier and Wullimann, 2008) and so, the situation observed in the shark is likely to occur in other vertebrates. Besides having a hypothalamic location, this TH-ir cell population seems to have a hypothalamic origin among different vertebrates. Except in reptiles and mammals, these cells emerge concurrently with those of the rostral hypothalamus in all vertebrates studied so far, supporting a conserved hypothalamic origin (Carrera et al., 2012). On the other hand, in *S. canicula*, *ScOtp* is expressed in the PM/PRM-like area from stage 25 onward (**Figure 3Q**) just before TH-ir cells emerge in the rostral posterior tuberculum (Carrera et al., 2012). Interestingly, in zebrafish, mutants lacking *Otp* expression in the hypothalamus also lack hypothalamic and posterior tubercular TH-ir groups (Ryu et al., 2007). Finally, at late stages in *S. canicula*, as in zebrafish and mouse (Ryu et al., 2007; Puelles et al., 2012), scattered *ScOtp*-expressing cells were observed outside the ventricular zone of the PM/PRM-like region entering the marginal zone of the RM-like and p3Tg (**Figures 4H** and **6B**), which support a PM/PRM-like origin for *ScOtp*-expressing cells in p3Tg.

Thus, it appears that, among different vertebrates, at least some populations of the RM-like and p3Tg emerge from hypothalamic domains expressing *Otp*, and that TH-ir cells of the RM-like domain send ascending projections to the telencephalon caudally to the IHB.

### Intrahypothalamic Boundary Identification

The updated prosomeric model proposes a secondary prosencephalon divided in two segments, hp2 and hp1, separated by the IHB. Both segments include a hypothalamic and a telencephalic counterpart (**Figure 6A**). This boundary is supported by (i) the existence of the anterior and retromammillary commissures in the roof and floor plates, respectively, (ii) the restricted expression of several genes to one or other segment, and (iii) the course of main tracts [medial forebrain bundle (mfb), lateral forebrain bundle (lfb) and fornix (fx)] separating both segments. Thus, there is a correlation among histogenetic data and fiber tract data. In fact, it was argued that the course of tracts is a powerful test for brain models since they are also guided by mechanisms related to those involved in histogenetic patterning (Puelles et al., 2012).

In the shark (**Figure 6B**), the IHB is also supported by (i) the existence of commissures in the alar and floor plates, (ii) the restricted expression of *ScShh* and *ScNkx2.1* in particular subdomains within either the rostral or the caudal segment, and (iii) the presence of different neurochemical populations of fibers, topologically homologue to those described in the model, which divide the hypothalamus in a caudal (peduncular, hp1) and a rostral (terminal, hp2) domain. Thus, we have tentatively defined an IHB-like based on genetic or histogenetic data and partial neurochemical or fiber tract data. We also discuss the congruence between the two kinds of data, when possible.

#### The Presence of Commissures

In the roof plate, according to the prosomeric model, the IHB ends caudal to the anterior commissure. This commissure has been identified in *S. canicula* by means of α–acetylated-tubulin immunoreactivity in early embryos (**Figure 3I**) and GFAP-immunoreactivity at late development (**Figure 4P**). In the floor plate, the IHB coincides with the border between MM and RM-like subdomains. In mouse, the IHB at this point can be associated with the retromammillary commissure (also known as Forel’s ventral tegmental commissure) where fx tracts cross at its rostral-most portion. This commissure seems to be continuous and indistinguishable from the prethalamic (p3Tg) commissure, a bit more caudally arranged, where fibers (partly p3Tg and partly from the raphe nuclei) cross (see Puelles et al., 2012). Based on 5-HT-ir tracts, we have identified in *S. canicula* a conspicuous commissure rostrally located with respect to the HDB, as predicted in the frame of the prosomeric model. A hypothalamic commissure has been also identified at this point in adult chondrichthyan specimens, which has been referred as postinfundibular commissure and extends through the ventral and rostro-caudal extension of the posterior tuberculum. This postinfundibular commissure presents differential rostro-caudal connectivity and no other commissures have been described at this point, closely resembling the scenario found in the mouse. While the rostral portion (or pars superior) connects hypothalamic cell masses, fibers of the tract of the saccus vasculossus decussate in the caudal part (or pars inferior; Smeets, 1998). Previous work on the shark reveals 5-HT-ir and GAD-ir fibers crossing in the rostral and caudal extension of this commissure, respectively (Sueiro et al., 2007; Carrera, 2008). It has been proposed that these 5-HT-ir fibers belong to 5-HT-ir cells of the posterior tuberculum (Sueiro et al., 2007; Carrera, 2008) although this fact has not been confirmed. However, in mammals, on which 5-HT-ir projecting cells are only located in the brainstem, a similar commissure has been described and referred as supramammillar commissure (Botchkina and Morin, 1993) which we assumed as equivalent to the prosomeric retromammillary commissure (Puelles et al., 2004). While it remains unclear whether the postinfundibular commissure of sharks is homologous to the retromammillary commissure of mouse, it appears that its location fit with the floor plate limit of the IHB (**Figure 6B**).

#### Correspondence to Histogenetic Domains

Either the expression of different genes (that would help identifying different histogenetic domains) or, accordingly, the course of the IHB through the telencephalon, has not been analyzed here. In the AHy, the prosomeric model proposes that the IHB can be delineated just caudal to the optic stalk, at the caudal border of expression of genes like *Six3*, *Neurog3*, *Six6*, *Nkx2.6* or de rostral border of *Tbr1*, *Uncx4.1*, *Sim1*, *Olig2*, *Foxb1*, *Nr5a1*, which is the same point whereby the mfb, lfb, and fx course (Shimogori et al., 2010; Morales-Delgado et al., 2011, 2014; Puelles et al., 2012; see below). In the BHy, the model proposes that the IHB can be delineated just caudal to the caudal border of genes as *Nkx2.1*, *Olig2*, *Foxb1* or *Nr5a1* or de rostral border of genes as *Lmx1b*, *Lhx5*, *Ptix2*, *Lhx1*, *Lhx6*, *Lhx9*, *Arx* or *Irx5*, which is the same point whereby the fx run (Shimogori et al., 2010; Morales-Delgado et al., 2011, 2014; Puelles et al., 2012). Therefore, the PPa, PSPa, RTu, PRM, and RM domains belong to hp1, while TPa, TSPa, Tu, PM, and MM domains belong to hp2 (**Figure 6A**).

In the AHy of *S. canicula*, as discussed above, no evidence for subdivisions along the rostro-caudal axis could be found on the basis of *ScOtp* or *ScDlx2/5* expression. In the BHy, two distinct territories could be inferred from *ScNkx2.1* and *ScShh* expressions, the MM-like (rostral, *ScNkx2.1*-expressing) and the RM-like (caudal, *ScShh* expressing) domains (**Figure 6B**), which is consistent with the predictions of the prosomeric model. According to the prosomeric model, the IHB can be delineated between both domains. The other compartments (Tu/RTu and PM/PRM) are presumably divided by the IHB, but any other gene among those used here serves as a caudal (hp1) or rostral (hp2) marker, except for* ScShh* in the Tu/RTu-like domain that, in *S. canicula* (but not in mouse) seems to be restricted to the rostral (hp2) domain.

#### Main Tracts Coursing the Chondrichthyan Alar and Basal IHB

In the mouse, the fx is the only tract coursing from the alar to the basal plate that additionally decussates in the hypothalamic floor plate by the retromammillary commissure. The mfb is also a transverse peduncular tract whose rostral border follows the IHB (Puelles et al., 2012). In chondrichthyans, a fx counterpart has not been successfully confirmed to date (Smeets, 1998). Only a counterpart of the mfb has been described, which is referred as the basal forebrain bundle or *fasciculus basalis telencephali* in chondrichthyans literature [the sot in zebrafish literature (Smeets, 1998; Carrera, 2008; Carrera et al., 2008, 2012; Puelles et al., 2012)]. Ascending and descending projections between the telencephalon and the superior and caudal part of the inferior lobes (which probably correspond to the lateral and caudal part of the Tu/RTu-like domain defined here) have been experimentally confirmed coursing through the mfb of different adult chondrichthyans (Smeets, 1998; Hofmann and Northcutt, 2008). These facts support the existence of tracts coursing caudally to a hypothetic IHB. Interestingly, Carrera et al. (2012) have identified TH-ir fibers ascending from the posterior tuberculum to the telencephalon through the mfb, as in other vertebrates (see above; Vernier and Wullimann, 2008; Carrera et al., 2012). We argued above that the TH-ir cells of the rostral posterior tuberculum previously described as belonging to p3Tg in sharks, probably belong to what we identified here as the RM-like compartment, and that the situation observed in the shark is likely to occur in other vertebrates. Thus, TH-ir fibers likely ascending from the RM-like to the telencephalon seem to course caudally to the IHB (see above), which additionally support the identification of the rostral border of hp1. Therefore, the presence of tracts in the rostral border of the RM-like domain parsimoniously fits with the predictions of the prosomeric model respect the course of fiber tracts in the rostral border of the hp1prosomere and caudally to a hypothetic IHB (**Figure 6B**). Of note, 5-HT-ir and GAD-ir cells have been also identified in the posterior tuberculum of the shark and other vertebrates. Whether ascending projections from this source coursed to the telencephalon has not been determined to date (Barale et al., 1996; Mueller et al., 2006; Carrera, 2008; Carrera et al., 2008; Lillesaar, 2011). However, these tracts are likely to join those mfb tracts that ascend to the telencephalon just caudal to the optic stalk, indirectly drawing the boundary among the Tu-like and RTu-like.

In the alar plate, 5-HT-ir, GAD-ir, TH-ir and GFPA-ir fibers of the mfb, arising from different points of the brain, have been observed ascending to the telencephalon by a common path just caudal to the optic stalk, which could correspond to the alar IHB (Carrera, 2008; Carrera et al., 2012, **Figures 4J,K,O**). The most conspicuous of these tracts were 5-HT-ir tracts recognizable at stage 30 (Carrera, 2008; Carrera et al., 2008, and **Figures 4I–L** in present work). This path is also followed by GFAP-ir processes which, besides, cross at the anterior commissure (**Figures 4O,P**). Since, as reported in mouse, they coursed just caudal to the optic stalk, we propose that these tracts in *S. canicula* course caudally to the IHB, separating the TPa and TSPa (hp2) from the PPa and PSPa (hp1; **Figure 6B**). While, with the markers used here, we cannot ascertain if this boundary in the AHy is also supported by histogenetic domains, these tracts appear to course through gaps of *ScDlx2/5* and *ScOtp* expression in the telencephalon (**Figures 4M,N**, respectively).

To summarize, our data support the conclusion that, in *S. canicula*, an IHB topologically homologous to that proposed by the updated prosomeric model, courses from the anterior commissure (in the telencephalon) to the postinfundibular commissure (in the hypothalamus) through the mfb. Note that, in contrast to mouse, the IHB in *S. canicula* can be traced by partial genetic and fiber tract evidences, since tracts coursing through the telencephalon to the floor plate have not been demonstrated. In the alar plate the mfb courses topologically caudal to the optic stalk and is known to be composed by different neurochemical systems arising from different points of the brain. In the basal plate the mfb is known to be composed, at least, of TH-ir fibers which arise upward from the rostral posterior tuberculum.

## Conclusion

We have revisited and reinterpreted the organization of the developing hypothalamus in a chondrichthyan model, *S. canicula*, within a prosomeric and histogenetic framework. These data reveal striking similarities with the organization described in the mouse by means of the updated prosomeric model (Puelles et al., 2012). In the AHy we have tentatively identified TPa/PPa-like, TSPa/PSPa-like histogenetic domains and their boundaries. In the BHy we have identified similar histogenetic domains to those observed in the mouse (Tu/RTu, PM/PRM, RM/MM-like). The fact that *ScShh* was downregulated in a large portion of the BHy and *ScDlx2/5* expression was much more restricted than in mouse have allowed us to identify different subdomains within the Tu/RTu-like area. Furthermore, we have identified an IHB separating terminal and peduncular portions of telencephalon and hypothalamus, as the model predicts, based partially on genetic and fiber tract data. Altogether, these data show that the prosomeric model in its latest version provides an adequate reference to describe the molecular organization of the catshark developing hypothalamus, thus highlighting the underlying unity of this complex anatomical structure across jawed vertebrates.

## Conflict of Interest Statement

The authors declare that the research was conducted in the absence of any commercial or financial relationships that could be construed as a potential conflict of interest.

## Acknowledgments

This work was supported by grants from the Spanish Dirección General de Investigación-FEDER (BFU2010- 15816), the Xunta de Galicia (10PXIB200051PR, CN 2012/237), European Community-Research Infrastructure Action under the FP7 “Capacities” Specific Programme (ASSEMBLE 227799), the Région Centre, Région Bretagne (EVOVERT grant number 049755; PEPTISAN project), National Research Agency (grant ANR-09-BLAN-026201), CNRS, Université d’Orléans and Université Pierre et Marie Curie. GNSD would like to thank Spanish SEPE for its funding support.

## Supplementary Material

The Supplementary Material for this article can be found online at: http://www.frontiersin.org/journal/10.3389/fnana.2015.00037/abstract

## ABBREVIATIONS

ABB: alar-basal boundary
ac: anterior commissure
AHy: alar hypothalamus
At: acroterminal region
AP: alar plate
BHy: basal hypothalamus
BP: basal plate
CAHy: caudal part of the alar hypothalamus
CBHy: caudal part of the basal hypothalamus
D: diencephalon
F: forebrain
FP: floor plate
HDB: hypothalamo-diencephalic boundary
MM: mammillary area
MTT: mammillo-tegmental tract
mz: marginal zone
os: optic stalk
p1: prosomere 1
p2: pallium 2
p2Tg: tegmental part of prosomere 2
p3: prosomere 3
p3Tg: tegmental part of prosomere 3
P: pallium
PM: perimammillary area
POA: preoptic area
PPa: peduncular paraventricular area
PRM: periretromammillary area
PSPa: peduncular subparaventricular area
PThE: prethalamic eminence
RAHy: rostral part of the alar hypothalamus
RAHy: rostral part of the alar hypothalamus
Rh: rhombencephalon
RM: retromammillary area
rmc: retromammillary commissure
RP: roof plate
RTu: retrotuberal domain
sot: supraoptic tract
Sp: subpallium
T: telencephalon
TPa: terminal paraventricular area
TPOC: tract of the postoptic commissure
TSPa: terminal subparaventricular area
Tu: tuberal domain
vz: ventricular zone.
