# Supplementary material for: Prosomeric organization of the hypothalamus in an elasmobranch, the catshark Scyliorhinus canicula
Source: Front Neuroanat. 2015 Apr 8;9:37. doi: 10.3389/fnana.2015.00037 (PMC4389657; doi:10.3389/fnana.2015.00037)
Supplement: Supplementary file 1 [file Table_1.DOCX]

| **Gene** | **Species** | **Accession number** |  | **Gene** | **Species** | **Accession number** |
| --- | --- | --- | --- | --- | --- | --- |
| **Foxg1 family** | | |  | **Otp family** | | |
| Foxg1 | *Homo sapiens* | ENST00000313071 |  | Otp | *Homo sapiens* | ENST00000306422 |
| Foxg1 | *Gallus gallus* | ENSGALT00000016016 |  | Otp | *Gallus gallus* | ENSGALT00000045360 |
| Foxg1a | *Lepisosteus oculatus* | ENSLOCT00000021844 |  | Otp | *Xenopus tropicalis* | ENSXETT00000037209 |
| Foxg1a | *Oryzias latipes* | ENSORLT00000013443 |  | Otp | *Latimeria chalumnae* | ENSLACT00000017800 |
| Foxg1a | *Danio rerio* | ENSDART00000104204 |  | Otpb | *Danio rerio* | ENSDART00000081249 |
| Foxg1a | *Latimeria chalumnae* | ENSLACT00000014849 |  | Otpb | *Oryzias latipes* | ENSORLT00000004034 |
| Foxg1c | *Danio rerio* | ENSDART00000139352 |  | Otpa | *Danio rerio* | ENSDART00000027268 |
| Foxg1c | *Lepisosteus oculatus* | ENSLOCT00000021866 |  | Otpa | *Lepisosteus oculatus* | ENSLOCT00000006966 |
| Foxg1c | *Latimeria chalumnae* | ENSLACT00000006889 |  | Otp | *Callorhinchus milii* | XM_007905390.1 |
| Foxg1b | *Lepisosteus oculatus* | ENSLOCT00000021496 |  |  |  |  |
| Foxg1b | *Oryzias latipes* | ENSORLT00000002602 |  |  |  |  |
| Foxg1b | *Danio rerio* | ENSDART00000013642 |  |  |  |  |
| Foxg1d | *Danio rerio* | ENSDART0000010250 |  |  |  |  |
| Foxg1b | *Latimeria chalumnae* | ENSLACT00000011232 |  |  |  |  |
| BF1 | *Branchiostoma floridae* | XM_002610184.1 |  |  |  |  |
|  |  |  |  |  |  |  |
| **Tbr1/Tbx21/Eomes family** | | |  | **Hedgehog family** | | |
| Tbr1 | *Homo sapiens* | ENST00000389554 |  | Shh | *Homo sapiens* | ENST00000297261 |
| Tbr1 | *Gallus gallus* | ENSGALT00000018128 |  | Shh | *Anolis carolinensis* | ENSACAT00000011075 |
| Tbr1 | *Xenopus tropicalis* | ENSXETT00000035364 |  | Shh | *Lepisosteus oculatus* | ENSLOCT00000015397 |
| Tbr1 | *Lepisosteus oculatus* | ENSLOCT00000010453 |  | Shh | *Oryzias latipes* | ENSORLT00000013116 |
| Tbr1 | *Oryzias latipes* | ENSORLT00000021054 |  | Shh | *Danio rerio* | ENSDART00000149395 |
| Tbr1b | *Danio rerio* | ENSDART00000006612 |  | Shh | *Danio rerio* | ENSDART00000056747 |
| Tbr1a | *Danio rerio* | ENSDART00000040180 |  | Ihh | *Homo sapiens* | ENST00000295731 |
| Tbx21 | *Homo sapiens* | ENST00000177694 |  | Ihh | *Anolis carolinensis* | ENSACAT00000005165 |
| Tbx21 | *Anolis carolinensis* | ENSACAT00000007062 |  | Ihh | *Xenopus tropicalis* | ENSXETT00000030370 |
| Tbx21 | *Oryzias latipes* | ENSORLT00000015260 |  | Ihh | *Oryzias latipes* | ENSORLT00000002066 |
| Tbx21 | *Danio rerio* | ENSDART00000013980 |  | Ihh | *Danio rerio* | ENSDART00000081660 |
| Tbx21 | *Lepisosteus oculatus* | ENSLOCT00000016470 |  | Ihh | *Danio rerio* | ENSDART00000081760 |
| eomes | *Homo sapiens* | ENST00000449599 |  | Ihh | *Oryzias latipes* | ENSORLT00000020230 |
| eomes | *Gallus gallus* | ENSGALT00000042658 |  | Ihh | *Lepisosteus oculatus* | ENSLOCT00000013349 |
| eomes | *Xenopus tropicalis* | ENSXETT00000043113 |  | Dhh | *Homo sapiens* | ENST00000266991 |
| eomes1 | *Oryzias latipes* | ENSORLT00000002879 |  | Dhh | *Anolis carolinensis* | ENSACAT00000015591 |
| eomesa | *Danio rerio* | ENSDART00000122257 |  | Dhh | *Xenopus tropicalis* | ENSXETT00000024437 |
| eomesb | *Danio rerio* | ENSDART00000008317 |  | Dhh | *Oryzias latipes* | ENSORLT00000009520 |
| eomesa | *Oryzias latipes* | ENSORLT00000006798 |  | Dhh | *Danio rerio* | ENSDART00000053871 |
| eomesa | *Lepisosteus oculatus* | ENSLOCT00000002821 |  | Dhh | *Lepisosteus oculatus* | ENSLOCT00000009356 |
| Tbr/Tbx21/  eomes | *Branchiostoma floridae* | XM_002589046.1 |  | Hh | *Branchiostoma floridae* | XM_002592059.1 |
|  |  |  |  |  |  |  |
| **Nkx2.1/Nkx2.4 family** | | | | | | |
| Nkx2.1 | *Homo sapiens* | ENST00000498187 |  | Nkx2.1a | *Danio rerio* | ENSDART00000007829 |
| Nkx2.1 | *Gallus gallus* | NM_204616.1 |  | Nkx2.4 | *Homo sapiens* | ENST00000351817 |
| Nkx2.1b | *Lepisosteus oculatus* | ENSLOCT00000017285 |  | Nkx2.4 | *Xenopus tropicalis* | ENSXETT00000050715 |
| Nkx2.1b | *Danio rerio* | ENSDART00000017493 |  | Nkx2.4a | *Danio rerio* | ENSDART00000110871 |
| Nkx2.1b | *Oryzias latipes* | ENSORLT00000010822 |  | Nkx2.1 | *Branchiostoma floridae* | AF077840.1 |
| Nkx2.1a | *Lepisosteus oculatus* | ENSLOCT00000020894 |  |  |  |  |

**Supplementary Table 1 : Accession numbers of the sequences retrieved fromEnsembl and Genbank databases and included in the phylogenetic reconstructions shown in Figure 1.**

For each gene family (bold letters), gene name, species and the corresponding accession number are successively indicated. Genbank submissions of the catshark sequences used in this study are underway.
